# A rapid multiplex PCR assay for species identification of Asian rice planthoppers (Hemiptera: Delphacidae) and its application to early-instar nymphs in paddy fields

**DOI:** 10.1371/journal.pone.0250471

**Published:** 2021-04-23

**Authors:** Toshihisa Yashiro, Sachiyo Sanada-Morimura

**Affiliations:** Division of Agro-Environment Research, Kyushu Okinawa Agricultural Research Center, National Agriculture and Food Research Organization, Koshi, Kumamoto, Japan; USDA Agricultural Research Service, UNITED STATES

## Abstract

Rice (*Oryza sativa* L.) is the main cereal crop in many Asian countries. The Asian rice planthoppers, *Nilaparvata lugens* (Stål) (brown planthopper), *Sogatella furcifera* (Horváth) (white-backed planthopper), and *Laodelphax striatellus* (Fallén) (small brown planthopper) (Hemiptera: Delphacidae), are the most economically important pests of rice. These three rice planthopper species often co-occur in the same paddy field. Traditionally, species identification of individuals of the three rice planthopper species has relied on morphological characters, but accurate discrimination of early-instar nymphs is very difficult, even for expert researchers. In this study, we developed a rapid one-step multiplex PCR assay using conserved and species-specific *5*.*8S*-*ITS2* rDNA gene primers for simultaneous identification of individuals of the three rice planthopper species. The multiplex PCR results showed that the three rice planthopper species could be identified accurately based on the length of the resultant amplicon, regardless of the individual developmental stage. Furthermore, we applied this assay for the first accurate quantification of early-instar nymphs of each rice planthopper species in paddy fields. Notably, we found that the species composition of early-instar nymphs cannot be extrapolated from that of adults. Thus, the multiplex PCR assay developed here facilitates detection of each rice planthopper species at the beginning of outbreaks in paddy fields.

## Introduction

Numerous insects migrate long distances between countries and continents [1, 2]. Some of them are serious agricultural pests, such as locusts, armyworms, and planthoppers [3–5]. The timing and magnitude of migration of these transboundary pests vary from year to year because their long-distance migrations occur under specific weather conditions [3–5]. Therefore, it is necessary to monitor the migration and subsequent population growth of such insect pests continuously for their management [6, 7]. Molecular methods, including PCR-based techniques and DNA sequencing, are useful for identifying species, strains, and source populations of migratory individuals and populations [8–11]. Furthermore, these methods should be cost-effective and easy to use for routine analysis of large numbers of samples [10, 12].

Rice (*Oryza sativa* L.) is the staple food for more than half of the world’s population and more than 90% of the world’s rice is produced in Asia [13]. The Asian rice planthoppers, *Nilaparvata lugens* (Stål) (brown planthopper), *Sogatella furcifera* (Horváth) (white-backed planthopper), and *Laodelphax striatellus* (Fallén) (small brown planthopper) (Hemiptera: Delphacidae), are the most economically important pests of rice [14–18]. They can cause serious yield losses both directly by removing phloem sap and indirectly by transmitting viral diseases throughout East, Southeast, and South Asia, resulting in billions of dollars of economic loss annually [14, 15].

In temperate areas of East Asia, including Japan, Korea, and most of China, two of the three rice planthopper species, *N*. *lugens* and *S*. *furcifera*, are unable to overwinter successfully. Nevertheless, colonization by these two species occurs in these areas annually, following long-distance migration from overwintering areas, including southern China and northern Vietnam [19]. Moreover, although *L*. *striatellus* can overwinter in temperate areas of East Asia, *L*. *striatellus* can also migrate overseas from eastern China to Japan and Korea [20]. Consequently, the abundance and composition of the three rice planthopper species in these temperate areas are influenced by the timing and magnitude of their immigration [21]. The three rice planthopper species often co-occur in the same paddy field [15, 22], where each of the three have developed resistance to various insecticides independently [23–25]. Furthermore, *N*. *lugens* is the most serious pest of rice in most situations, while the other two rice planthopper species are normally less serious pests than *N*. *lugens* [16, 21]. Thus, the detection of each rice planthopper species in paddy fields is necessary for their efficient management.

Traditionally, species identification of individuals of rice planthoppers has relied on morphological characters [22, 26]. However, accurate discrimination of early-instar nymphs of the three rice planthopper species by conventional morphological identification methods is very difficult, even for expert researchers [27]. This hampers detection of the three rice planthopper species at the beginning of outbreaks in paddy fields. By contrast, molecular identification is not limited by the developmental stage of the target species. Although molecular diagnostic methods for rice planthopper species have been developed, they are time consuming and/or expensive—multiple PCR assays with specific primer pairs [28] or a single multiplex PCR assay with fluorescent dye-labeled primers and capillary electrophoresis are needed for species identification for each sample [29].

In this study, we developed a new molecular identification method for the three rice planthopper species, based on a single multiplex PCR with non-fluorescently labeled primers and agarose gel electrophoresis. Furthermore, we applied this identification method for the first accurate quantification of early-instar nymphs of each rice planthopper species in paddy fields.

## Materials and methods

### Rice planthoppers

Adult rice planthoppers were collected in various years and locations, and identified morphologically to the species level according to Ref [30] (NL1–NL10, SF1–SF10, and LS1–LS10 in S1 Table). Immediately after field collection, the adults of NL7–NL10, SF7–SF10, and LS7–LS10 were preserved at −25°C until use (i.e., wild-caught adults). After rearing individuals of NL1–NL6, SF1–SF6, and LS1–LS6 separately for several generations using rice seedlings at 25°C under a 16 h light:8 h dark photoperiod, the adults and nymphs were preserved at −25°C until further use (i.e., laboratory-reared adults and laboratory-reared nymphs).

### Multiplex PCR primer design

**Fig 1:**
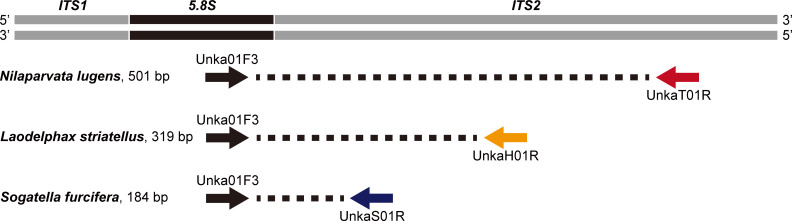
Schematic overview of conserved rice planthopper primers and species-specific primers targeting the partial *5*.*8S* and *ITS2* rDNA sequences, and corresponding PCR products allowing the identification of *Nilaparvata lugens*, *Laodelphax striatellus*, and *Sogatella furcifera* in a multiplex PCR. The primer sequences are listed in Table 1.

One male adult was randomly chosen from each of NL1–NL10, SF1–SF10, and LS1–LS10 (S1 Table). The sequences of the three rice planthopper species collected from Asian countries (Japan, China, Vietnam, Philippines, and Papua New Guinea) available in GenBank (accession numbers KC660120–KC660122 and AB625596–AB625609) were also used to design multiplex PCR primers. DNA was extracted and purified in accordance with the Chelex protocol modified from Ref [31]. Legs of planthoppers were individually ground in 10% w/v Chelex-100 (Bio-Rad, Hercules, CA, USA) in nuclease-free water using a pestle for several minutes, incubated at 95°C for 15 min, and centrifuged at 7,000 rpm for 1 min; the supernatant was used for PCR amplification after the DNA concentration was measured on a Qubit instrument (Thermo Fisher Scientific, Waltham, MA, USA). A fragment of *5*.*8S*-*ITS2*-*28S* rDNA was amplified by PCR using the primer set, ITS2F (5′-TGTGAACTGCAGGACACATGAA-3′) and ITS2R (5′-ATGCTTAAATTTAGGGGGTAGTC-3′) [32]. PCR was conducted using a My Cycler™ Thermal Cycler (Bio-Rad, Hercules, CA, USA) with a total reaction volume of 50 μL, containing 5 μL of 10× Ex-Taq^®^ buffer (Mg^2+^ plus) (TaKaRa Bio, Shiga, Japan), 4 μL of dNTPs (2.5 mM each), 2.5 μL of each primer (10 μM), 0.25 μL of Ex-Taq^®^ DNA polymerase (TaKaRa Bio), and 2 μL of DNA template. The PCR cycling conditions consisted of an initial denaturation at 95° C for 3 min; followed by 40 cycles of denaturing at 94°C for 40 s, annealing at 56°C for 40 s, and extension at 72°C for 60 s; then a final extension at 72°C for 10 min. PCR products were gel-purified using a Wizard^®^ SV Gel and PCR Clean-Up System (Promega, Madison, WI, USA) according to the manufacturer’s instructions, and sequenced by Eurofins Genomics (Tokyo, Japan) in both directions using the PCR primers. Consensus sequences were produced manually using BioEdit 7.0.4.1 [33]. Primers were designed manually based on sequence alignments in ClustalX 2.1 [34]. Four *5*.*8S*-*ITS2* rDNA gene primers (i.e., a single conserved forward primer [Unka01F3 in Table 1] and three species-specific reverse primers [UnkaT01R, UnkaS01R, and UnkaH01R in Table 1]) were designed for a multiplex PCR to generate amplicons of three different lengths unique to each of the three rice planthopper species (501 bp for *N*. *lugens*, 319 bp for *S*. *furcifera*, and 184 bp for *L*. *striatellus*) (Fig 1). The *5*.*8S*-*ITS2*-*28S* rDNA gene sequences obtained in this study were deposited in the DDBJ/EMBL/GenBank nucleotide sequence databases under the accession numbers MT950726–MT950755.

**Table 1:** Primers designed for the multiplex PCR assay in this study.

Primer name	Target species	Primer sequence (5’→3’)	Length	Tm	Product size
			(bp)	(°C)	(bp)
Universal forward primer					
Unka01F3	Rice planthoppers	ACATTTHGAACGCACRTTGC	20	53	-
Species-specific reverse primers					
UnkaT01R	*Nilaparvata lugens*	CTACACACATTTGGTACACAC	21	54	501
UnkaS01R	*Sogatella furcifera*	TAGGACGAGCCATTGCTTAG	20	55	184
UnkaH01R	*Laodelphax striatellus*	GGAAGGTGTTCATTAATCGGC	21	56	319

### Validation of the multiplex PCR primers

To validate the multiplex PCR primers, we used 10 wild-caught adults (5 males and 5 females) randomly chosen from each of NL7–NL10, SF7–SF10, and LS7–LS10 (S1 Table), 10 laboratory-reared adults (5 males and 5 females) randomly chosen from each of NL2–NL4, SF2–SF4, and LS3–LS5 (S1 Table), and 32 laboratory-reared nymphs (16 last-instar nymphs and 16 first-instar nymphs) randomly chosen from each of NL1, NL5, NL6, SF1, SF5, SF6, LS1, LS2, and LS6 (S1 Table). Legs of adults and last-instar nymphs and whole bodies of first-instar nymphs were individually ground in Chelex-100 resin solution (Bio-Rad, Hercules, CA, USA). DNA was extracted and purified in accordance with the Chelex protocol described above. The DNA concentrations of the extracts were measured on a Qubit instrument (Thermo Fisher Scientific, Waltham, MA, USA). PCR was conducted using a My Cycler™ Thermal Cycler (Bio-Rad, Hercules, CA, USA) with a total reaction volume of 20 μL, containing 2 μL of 10× Ex-Taq^®^ buffer (Mg^2+^ plus) (TaKaRa Bio, Shiga, Japan), 1.6 μL of dNTPs (2.5 mM each), 0.9 μL of the conserved forward primer (i.e., Unka01F3 in Table 1) (10 μM), 0.3 μL of each of the three species-specific reverse primers (i.e., UnkaT01R, UnkaS01R, and UnkaH01R in Table 1) (10 μM), 0.1 μL of Ex-Taq^®^ DNA polymerase (TaKaRa Bio, Shiga, Japan), and 1 μL of DNA template. The PCR cycling conditions consisted of an initial denaturation at 95°C for 1 min; followed by 30 cycles of denaturing at 94°C for 30 s, annealing at 56°C for 30 s, and extension at 72°C for 30 s; then a final extension at 72°C for 5 min. The PCR products were separated by 3% agarose gel (Agarose S; Nippon Gene, Tokyo, Japan) electrophoresis with 1× TAE buffer, stained with SAFELOOK™ Load-Green (Wako, Osaka, Japan), and visualized by illumination with blue LED light.

As a preliminary analysis, we also applied the multiplex PCR assay to individuals of congeners of *N*. *lugens* (i.e., *N*. *muiri*) and *S*. *furcifera* (i.e., *S*. *kolophon*). We collected 3 adults (2 males and 1 females) of *N*. *muiri* from Nagasaki, Japan, and 10 adults (5 males and 5 females) of *S*. *kolophon* from Kumamoto, Japan. The multiplex PCR assay was performed as described above. To verify the integrity of the DNA used in this experiment, samples were amplified by PCR using the *COI* universal primers, Calvin (5′-GGRAARAAWGTTAARTTWACTCC-3′) [35] and C1-J-2195 (5′-TTGATTTTTTGGTCAYCCWGAAGT-3′) [36]. PCR was conducted using a My Cycler™ Thermal Cycler (Bio-Rad, Hercules, CA, USA) with a total reaction volume of 20 μL, containing 2 μL of 10× Ex-Taq^®^ buffer (Mg^2+^ plus) (TaKaRa Bio, Shiga, Japan), 1.6 μL of dNTPs (2.5 mM each), 1 μL of each primer (10 μM), 0.1 μL of Ex-Taq^®^ DNA polymerase (TaKaRa Bio, Shiga, Japan), and 1 μL of DNA template. The PCR cycling conditions consisted of 40 cycles of denaturing at 94°C for 1 min, annealing at 45°C for 1 min, and extension at 72°C for 1 min; then a final extension at 72°C for 10 min. The PCR products were analyzed by agarose gel electrophoresis as described above.

### Quantification of early-instar nymphs in paddy fields

To quantify the abundance of each rice planthopper species in paddy fields, we sampled planthoppers using a standard sticky board method (modified from Ref [37]) in two experimental paddy fields (A and B; planting information is provided in S2 Table) at the Kyushu Okinawa Agricultural Research Center (Kumamoto, Japan), approximately one month after rice-planting (paddy field A, June 15 2020; paddy field B, July 9 2020). A rectangular plastic board (18 × 25 cm) coated with a sticky substance (Kinryu^®^ Spray; SDS Biotech, Tokyo, Japan) was held horizontally on one side of a rice hill at the bottom (about 2 cm above the paddy water), and the rice hill was beaten twice by hand to cause planthoppers to drop onto the sticky board. This procedure was repeated 20 times to collect planthoppers from 20 rice hills with one sticky board; three boards were used for each paddy field. The planthoppers captured on the boards were transported to the laboratory, and individual planthoppers were classified according to their developmental stage (i.e., early-instar nymph [instars I and II], mid-instar nymph [instars III and IV], late-instar [last instar] nymph [instar V], or adult). For adults, individual planthoppers were morphologically identified to the species level according to Ref [30]. For nymphs, individual planthoppers were identified as one of three species using the multiplex PCR assay. The nymph and adult species composition in each paddy field was compared using a chi-squared test (JMP 13; SAS Institute, Cary, NC, USA). The nymph and adult species compositions between paddy fields were also compared using a chi-squared test (JMP 13; SAS Institute, Cary, NC, USA), respectively.

## Results

### Validation of the multiplex PCR assay

**Fig 2:**
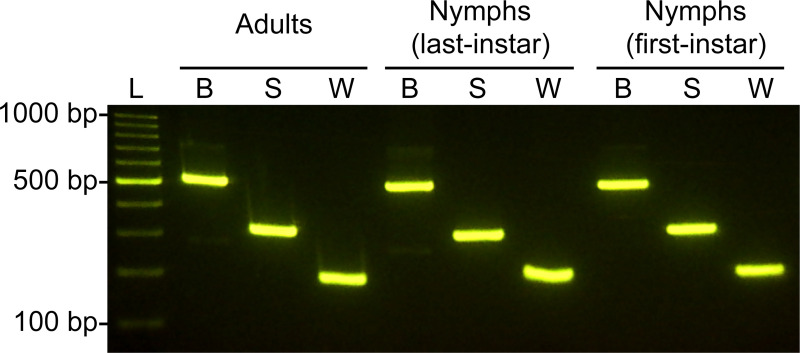
Detection of the three rice planthopper species by a multiplex PCR assay. L, 100 bp DNA ladder; B, *Nilaparvata lugens*; S, *Laodelphax striatellus*; W, *Sogatella furcifera*.

A total of 498 individuals of rice planthoppers, comprising 210 adults, 144 last-instar nymphs, and 144 first-instar nymphs, were tested using the multiplex PCR assay. The multiplex PCR primer set yielded a single clear band for each of the tested individuals of the three rice planthopper species with 100% identification accuracy (*N*. *lugens*, *n* = 166; *S*. *furcifera*, *n* = 166; *L*. *striatellus*, *n* = 166) (Fig 2).

In addition, 3 adults of *N*. *muiri* and 10 adults of *S*. *kolophon* were tested using the multiplex PCR assay. The multiplex PCR primer set generated no clear band for any tested individual of these two planthopper species (S1 Fig). The integrity of the DNA was verified by PCR using the *COI* universal primers (S1 Fig).

### Quantification of early-instar nymphs in paddy fields

**Fig 3:**
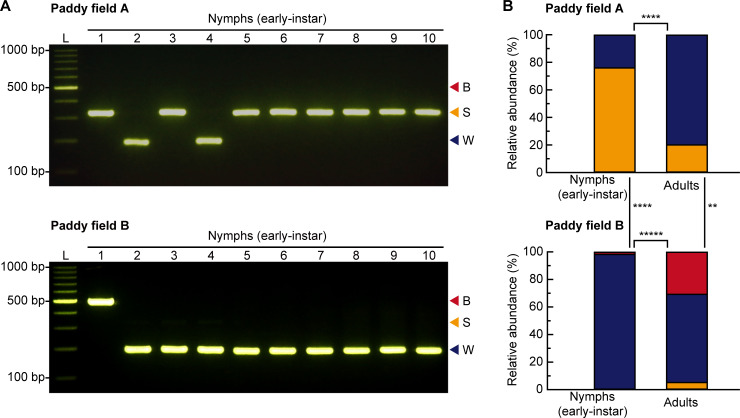
Quantification of rice planthopper species in paddy fields approximately one month after rice-planting. (A) Example gel electrophoresis results of multiplex PCR assays of individual early-instar nymphs from paddy fields A (top) and B (bottom). L, 100 bp DNA ladder; B, *Nilaparvata lugens*; S, *Laodelphax striatellus*; W, *Sogatella furcifera*. (B) Species composition of early-instar nymph and adult rice planthoppers (*N*. *lugens* [red bars], *S*. *furcifera* [blue bars], and *L*. *striatellus* [yellow bars]) in paddy fields A (early-instar nymphs, *n* = 54; adults, *n* = 20) (top) and B (early-instar nymphs, *n* = 107; adults, *n* = 39) (bottom). **, *P* < 0.01; ****, *P* < 0.0001; *****, *P* < 0.00001 (chi-squared test).

All collected adult individuals (paddy field A, *n* = 20; paddy field B, *n* = 37) were morphologically identified as three species (i.e., *N*. *lugens*, *S*. *furcifera*, and *L*. *striatellus*). All collected nymph individuals (paddy field A, *n* = 54; paddy field B, *n* = 107) were early-instar nymphs, identified as one of three species of rice planthoppers by the multiplex PCR assay (Fig 3A). The species composition of rice planthoppers differed significantly between early-instar nymphs and adults in each paddy field (chi-squared test: paddy field A, *P* < 0.0001; paddy field B, *P* < 0.00001) (Fig 3B). Moreover, the species compositions of both early-instar nymphs and adults differed significantly between paddy fields (chi-squared test: early-instar nymphs, *P* < 0.0001; adults, *P* < 0.01) (Fig 3B).

## Discussion

We developed a new molecular identification method for the most economically important pests of rice, the Asian rice planthoppers (i.e., *N*. *lugens*, *S*. *furcifera*, and *L*. *striatellus*). Our multiplex PCR assay enables robust and accurate species identification of Asian rice planthoppers, irrespective of developmental stage, sex, and collection date and locality (Fig 2). This assay significantly reduces the detection time and costs compared to previous molecular identification methods for Asian rice planthoppers [28, 29].

For the first accurate quantification of early-instar nymphs of the three rice planthopper species in paddy fields, we used a multiplex PCR assay developed in this study. Because the quantification was performed approximately one month after rice-planting in the two paddy fields, all collected individuals were early-instar nymphs or adults (i.e., the initial stage of occurrence of rice planthopper nymphs in paddy fields). All collected individuals of early-instar nymphs were successfully identified as one of three species by the multiplex PCR assay (Fig 3A). Thus, our multiplex PCR assay enables accurate detection of the three rice planthopper species, even at the beginning of outbreaks in paddy fields.

It is generally assumed that the species composition of rice planthopper early-instar nymphs can be roughly estimated from that of adults in the same paddy field. Indeed, pest control advisors and farmers often evaluate the occurrence of early-instar nymphs by referring to the proportion of adults occurring at that time and use pest control strategies against the estimated most abundant planthopper species because it is difficult to discriminate early-instar nymphs of the three species [27]. However, the rice planthopper species composition of early-instar nymphs was significantly different from that of adults, even within the same paddy field (Fig 3B). The lower proportion of individuals of *S*. *furcifera* in early-instar nymphs than in adults in paddy field A (Fig 3B) could be explained by ovicidal resistance which is a natural rice defense mechanism primarily against *S*. *furcifera* [38]. The higher proportion of individuals of *N*. *lugens* in adults than in early-instar nymphs in paddy field B (Fig 3B) could be explained by multiple immigration events of *N*. *lugens* from mainland China to Japan in July 2019 [39]. Hence, the planthopper species composition of early-instar nymphs cannot be extrapolated from that of adults. Likewise, the species composition of early-instar nymphs or larvae might not be simply extrapolated from that of adults of other insect pests of rice, such as the green rice leafhoppers, *Nephotettix* spp. (Hemiptera: Cicadellidae), that often co-occur in the same paddy field [40, 41] and can develop biotypes that respond differently to rice resistance [42]. Our surveys also showed a significant difference in the species composition of rice planthopper early-instar nymphs, as well as that of adults, between the two paddy fields (Fig 3B). This might be explained by the differences in rice variety or planting date between the two paddy fields (S2 Table). Future studies are needed to investigate in detail what factors are associated with the occurrence of each rice planthopper species in paddy fields; in such studies, the multiplex PCR assay developed in this study will be useful.

In addition, our multiplex PCR assay was applied to congeners of *N*. *lugens* and *S*. *furcifera* (i.e., *N*. *muiri* and *S*. *kolophon*), both of which are not pests of rice [26], and no clear bands were detected (S1 Fig). Although this study focused only on the three rice planthopper species, this multiplex PCR method could also be useful for detecting individuals of the three rice planthopper species from samples containing other planthopper species (e.g., light-trap samples) by redesigning the species-specific primers as needed.

## Conclusion

Uncontrollable outbreaks of Asian rice planthoppers led to a crisis in paddy ecosystems [14–18]. The multiplex PCR assay presented here provides a rapid, reliable, cost-effective method for species identification of individuals of the three rice planthopper species, irrespective of developmental stage. In this study, the multiplex PCR assay revealed a discrepancy in species composition between early-instar nymphs and adults in the same paddy field. Thus, this assay would contribute to the development of forecasting systems for rice planthopper outbreaks through better understanding of the migration and subsequent population growth of Asian rice planthoppers.

## Supporting information

S1 Fig Preliminary use of a multiplex PCR assay for congeneric species of rice planthoppers. L, 100 bp DNA ladder; NM, *Nilaparvata muiri*; SK, *Sogatella kolophon*.
(PDF)

S1 Table Rice planthopper samples sequenced in this study. (DOCX)

S2 Table Planting information of the paddy fields used in this study. (DOCX)

S1 Dataset Dataset for the validation of the multiplex PCR primers. (XLSX)

S2 Dataset Dataset for the quantification of early-instar nymphs in paddy fields. (XLSX)

S1 Raw images Uncropped gel images used to generate Figs 2, 3A, and S1 Fig. (PDF)
