# Supplementary figures and images for: A rapid multiplex PCR assay for species identification of Asian rice planthoppers (Hemiptera: Delphacidae) and its application to early-instar nymphs in paddy fields
Source: PLoS One. 2021 Apr 23;16(4):e0250471. doi: 10.1371/journal.pone.0250471 (PMC8064520; doi:10.1371/journal.pone.0250471)

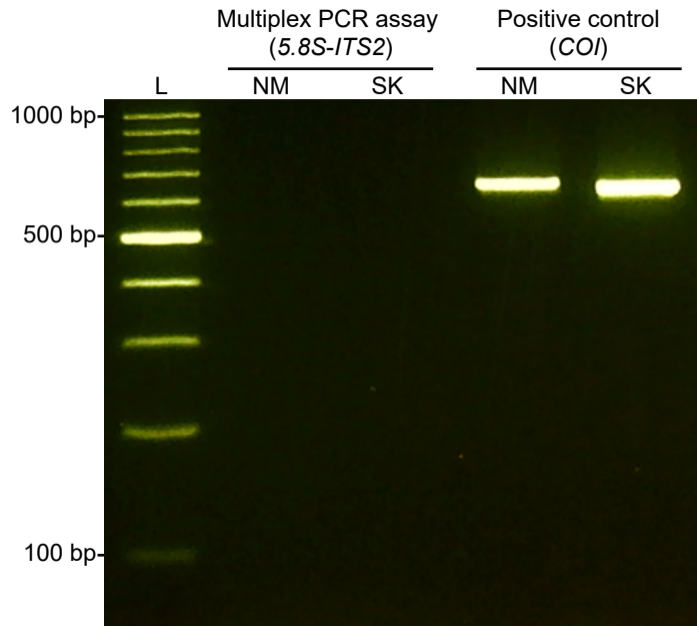

Supplement: S1 Fig — L, 100 bp DNA ladder; NM, Nilaparvata muiri; SK, Sogatella kolophon. (PDF) [file pone.0250471.s001.pdf]

Raw image data related to Fig 2

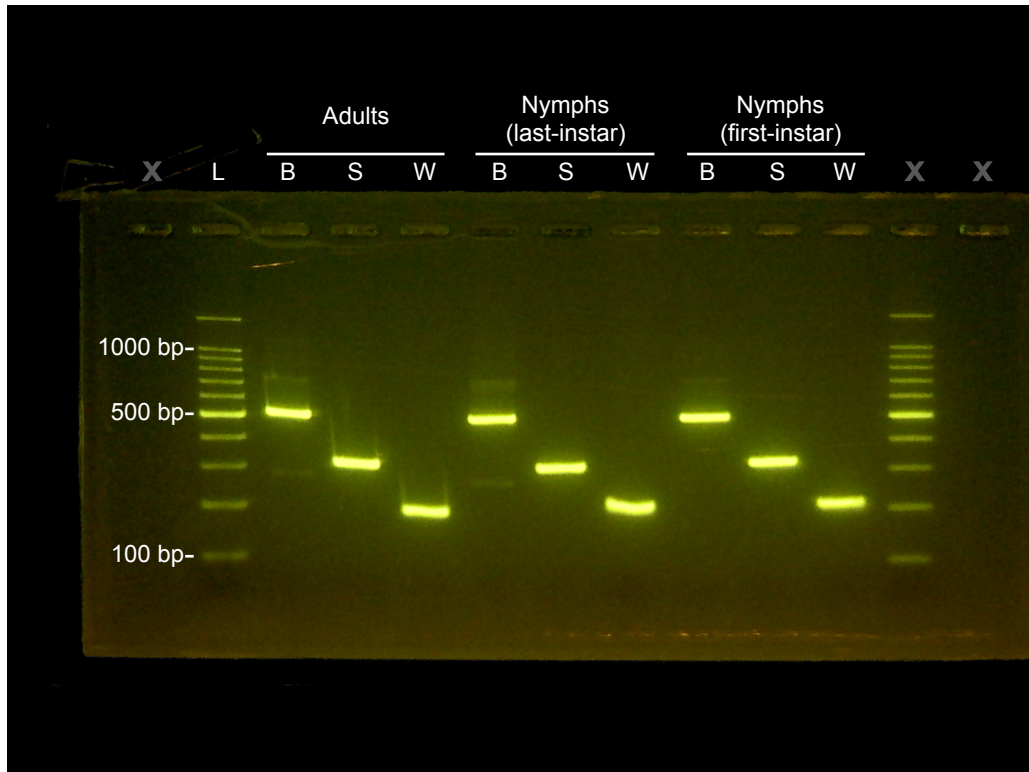

# Raw image data related to Fig 3A

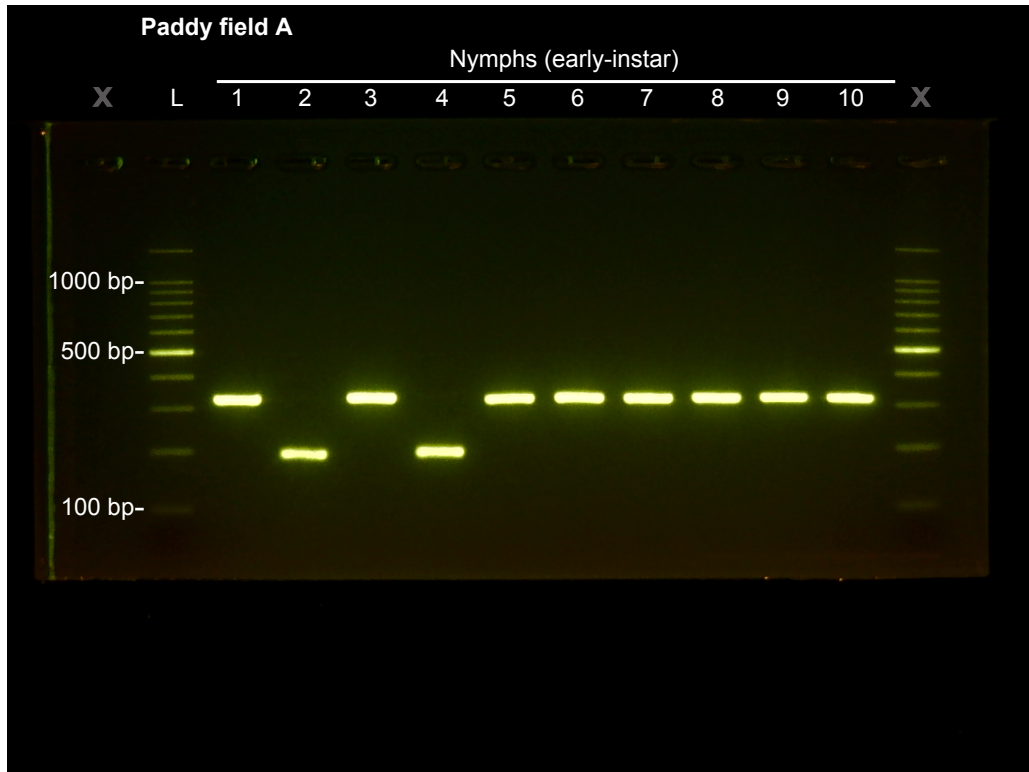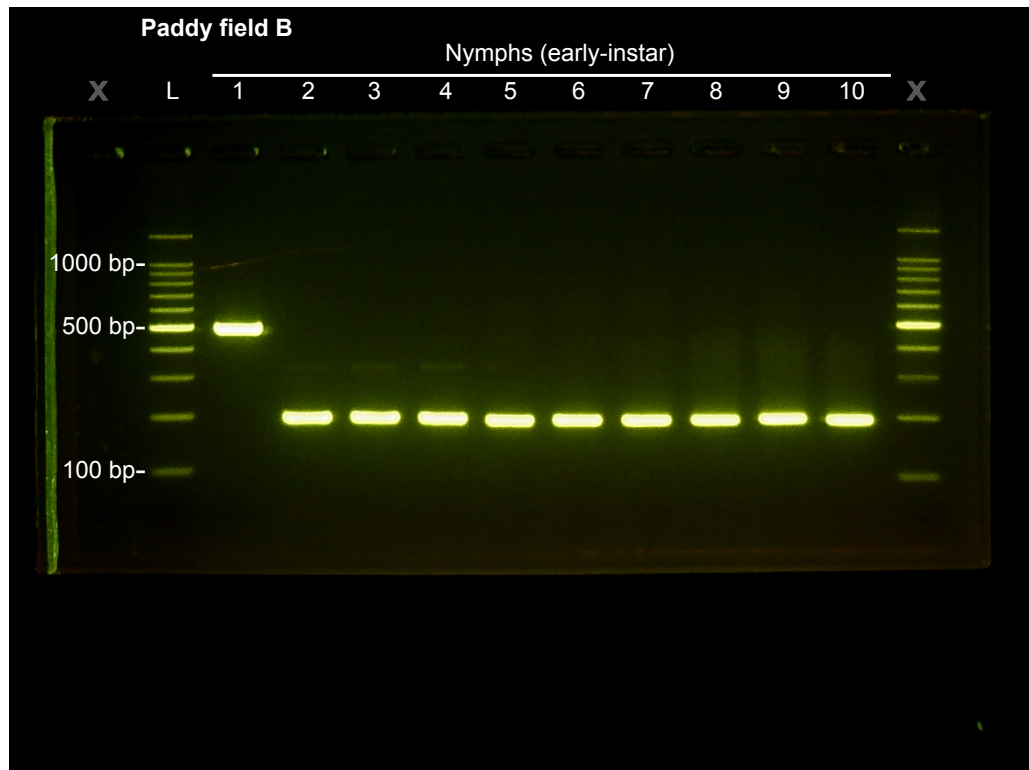

Raw image data related to S1 Fig

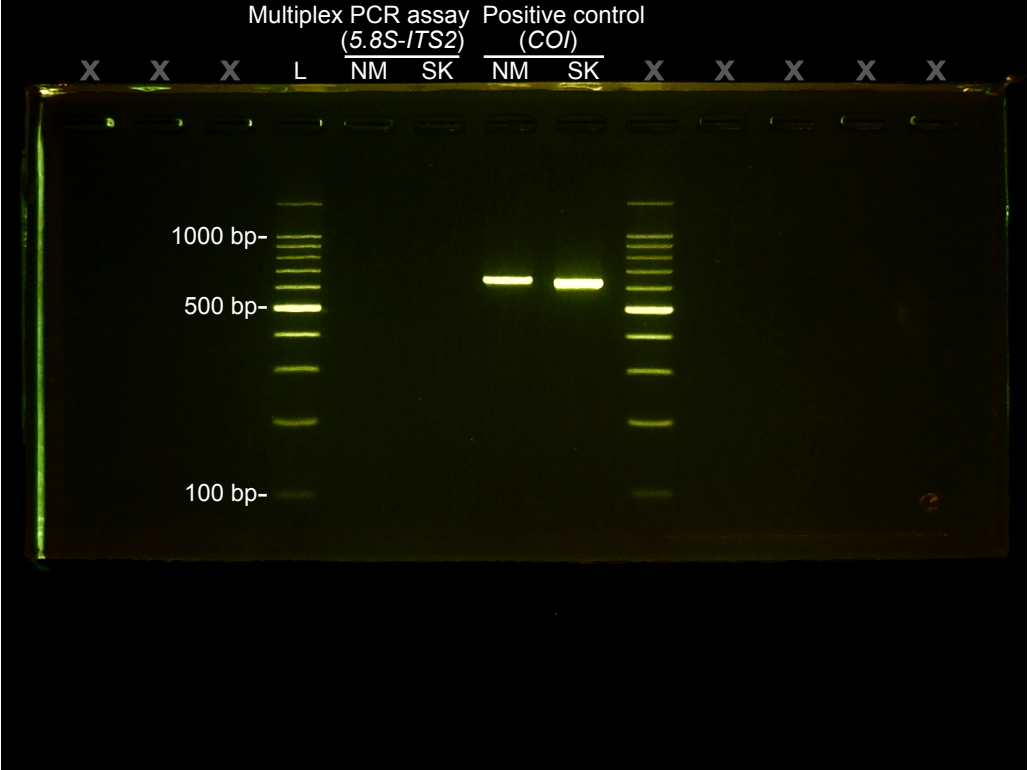

Supplement: S1 Raw images — (PDF) [file pone.0250471.s006.pdf]
