# Supplementary material for: A rapid multiplex PCR assay for species identification of Asian rice planthoppers (Hemiptera: Delphacidae) and its application to early-instar nymphs in paddy fields
Source: PLoS One. 2021 Apr 23;16(4):e0250471. doi: 10.1371/journal.pone.0250471 (PMC8064520; doi:10.1371/journal.pone.0250471)
Supplement: S1 Table — (DOCX) [file pone.0250471.s002.docx]

**S1 Table. Rice planthopper samples sequenced in this study.**

| **Population**  **/ Strain** | **Collection date** | **Location** | **GenBank no.**  **(*5.8S-ITS2-28S* rDNA)** |
| --- | --- | --- | --- |
| *Nilaparvata*  *lugens* |  |  |  |
| NL1 | 1966 | Hadano, Kanagawa, Honshu, Japan | MT950726 |
| NL2 | 1987 | Izumo, Shimane, Honshu, Japan | MT950727 |
| NL3 | 1989 | Chikugo, Fukuoka, Kyushu, Japan | MT950728 |
| NL4 | 17 August 2016 | Minamisatsuma, Kagoshima, Kyushu, Japan | MT950729 |
| NL5 | 10 October 2017 | Koshi, Kumamoto, Kyushu, Japan | MT950730 |
| NL6 | 11 September 2018 | Minamisatsuma, Kagoshima, Kyushu, Japan | MT950731 |
| NL7 | 28 August 2019 | Koshi, Kumamoto, Kyushu, Japan | MT950732 |
| NL8 | 5 September 2019 | Nagasaki, Nagasaki, Kyushu, Japan | MT950733 |
| NL9 | 14 October 2019 | Sakaide, Kagawa, Shikoku, Japan | MT950734 |
| NL10 | 15 October 2019 | Yamaguchi, Yamaguchi, Honshu, Japan | MT950735 |
|  |  |  |  |
| *Sogatella*  *furcifera* |  |  |  |
| SF1 | 1989 | Chikugo, Fukuoka, Kyushu, Japan | MT950736 |
| SF2 | 1999 | Koshi, Kumamoto, Kyushu, Japan | MT950737 |
| SF3 | 2 July 2012 | Koshi, Kumamoto, Kyushu, Japan | MT950738 |
| SF4 | 16 July 2014 | Koshi, Kumamoto, Kyushu, Japan | MT950739 |
| SF5 | 10 August 2017 | Koshi, Kumamoto, Kyushu, Japan | MT950740 |
| SF6 | 27 July 2018 | Minamisatsuma, Kagoshima, Kyushu, Japan | MT950741 |
| SF7 | 28 August 2019 | Koshi, Kumamoto, Kyushu, Japan | MT950742 |
| SF8 | 5 September 2019 | Nagasaki, Nagasaki, Kyushu, Japan | MT950743 |
| SF9 | 14 October 2019 | Sakaide, Kagawa, Shikoku, Japan | MT950744 |
| SF10 | 15 October 2019 | Yamaguchi, Yamaguchi, Honshu, Japan | MT950745 |
|  |  |  |  |
| *Laodelphax*  *striatellus* |  |  |  |
| LS1 | 1967 | Tokyo, Honshu, Japan | MT950746 |
| LS2 | 2008 | Minamisatsuma, Kagoshima, Kyushu, Japan | MT950747 |
| LS3 | 6 September 2010 | Ishigaki, Okinawa, Ryukyu Islands, Japan | MT950748 |
| LS4 | 21 September 2010 | Furukawa, Miyagi, Honshu, Japan | MT950749 |
| LS5 | 13 September 2018 | Kodera, Hyogo, Honshu, Japan | MT950750 |
| LS6 | 24 October 2018 | Tohma, Hokkaido, Hokkaido, Japan | MT950751 |
| LS7 | 28 August 2019 | Koshi, Kumamoto, Kyushu, Japan | MT950752 |
| LS8 | 5 September 2019 | Nagasaki, Nagasaki, Kyushu, Japan | MT950753 |
| LS9 | 14 October 2019 | Sakaide, Kagawa, Shikoku, Japan | MT950754 |
| LS10 | 15 October 2019 | Yamaguchi, Yamaguchi, Honshu, Japan | MT950755 |
