# Supplementary material for: A rapid multiplex PCR assay for species identification of Asian rice planthoppers (Hemiptera: Delphacidae) and its application to early-instar nymphs in paddy fields
Source: PLoS One. 2021 Apr 23;16(4):e0250471. doi: 10.1371/journal.pone.0250471 (PMC8064520; doi:10.1371/journal.pone.0250471)
Supplement: S2 Table — (DOCX) [file pone.0250471.s003.docx]

**S2 Table. Planting information of the paddy fields used in this study.**

| **Paddy field** | **GPS** | **Rice variety** | **Planting date** |
| --- | --- | --- | --- |
| A | N32.87381 E130.74035 | Koshihikari | 11 May 2020 |
| B | N32.87429 E130.74105 | Nikomaru | 15 June 2020 |
